# Aposematic Coloration of Moths Decreases Strongly along an Elevational Gradient in the Andes

**DOI:** 10.3390/insects12100903

**Published:** 2021-10-03

**Authors:** Konrad Fiedler, Gunnar Brehm

**Affiliations:** 1 Department of Botany & Biodiversity Research, University of Vienna, Rennweg 14, 1030 Vienna, Austria; 2 Institut für Zoologie und Evolutionsforschung mit Phyletischem Museum, Friedrich-Schiller Universität Jena, 07743 Jena, Germany; gunnar.brehm@uni-jena.de

**Keywords:** defensive coloration, elevational gradient, tropical Andes, tiger moths, predation risk

## Abstract

**Simple Summary:**

Certain moths defend themselves with toxic substances, and they show this to predators with bright and contrasting coloration. At high elevations, fewer birds, bats and other predators are present that feed on these insects. We therefore expected a decreasing proportion of colorful tiger and lichen moths with increasing elevation. Our study was carried out in forests between 1040 and 2670 m in the Ecuadorian Andes. We scored all 353 observed species according to their warning coloration, and whether they mimic bees and wasps or potentially poisonous beetles. We also measured forewing length of all species. From these data community-weighted means were calculated and related to the elevation of their collection sites. As predicted, the communitywide incidence of all three defensive traits decreased substantially from tall premontane forest to open upper montane forest. In parallel, moth size increased with elevation. Moreover, the systematic composition of tiger and lichen moth assemblages changed substantially. These findings support the idea that different selection regimes favor warning coloration at lower sites with higher predation pressure, while cryptic appearance is more advantageous at higher elevations.

**Abstract:**

On tropical mountains, predation pressure decreases with elevation. Accordingly, one expects an elevational decay in the prevalence of costly defensive traits such as aposematic coloration. Using light-trap catches of Arctiinae moths (353 species, 4466 individuals), assembled along a forested gradient in the megadiverse tropical Andes of southern Ecuador, we show that the incidence of aposematic coloration decreases strongly between 1040 and 2670 m asl. While over 60% of Arctiinae moths were warningly colored at lowest sites, this fraction decreased to less than 20% in montane forest, yet increased slightly again at the highest sites in the very open *Purdiaea nutans* forest. In parallel, the incidence of hymenopteran mimics and of species that mimic chemically defended beetles decreased with elevation. Hymenopteran mimics accounted for less than 5% of Arctiinae moths at sites above 2100 m, and beetle mimics were essentially lacking at high elevations. These patterns coincide with a change in gross taxonomic composition of Arctiinae ensembles and with an increase in average body size towards higher elevations. Representatives of Euchromiina and Ctenuchina became scarce with altitude, whereas the prevalence of Lithosiinae increased. Our findings suggest that the variable selective pressures along the elevational gradient favor warning coloration primarily at lower sites, whereas cryptic appearance of adult moths dominates in the tropical upper montane forest.

## 1. Introduction

Predation pressure is an important selective force in all food webs. The co-evolutionary arm’s race between prey and predators fuels the rise and continual refinement of defensive traits in prey organisms, as well as the evolution of counterstrategies to circumvent these defenses on part of the predators [1,2]. However, defensive characteristics usually come with costs. For example, resources need to be allocated between defense and reproduction, sequestered plant secondary compounds need to be handled, modified and stored in the body, and certain traits may be advantageous against some predators, but not against others. Hence, for each species the balance between the costs and benefits of expressing defensive traits needs to be met [3]. In addition, potentially suitable secondary plant compounds to be sequestered for defense are not equally available in all host plants. Accordingly, potential prey species in a food web vary in their level of defense. Some species may invest proportionally more in defense, whereas others rather attempt to avoid interactions with their enemies or follow other alternative strategies for survival. If predation pressure varies in a predictable manner along an ecological gradient, one therefore expects to see this mirrored by gradients in the prevalence of defensive traits, across species, at the community level.

Elevational gradients are a fruitful paradigm in ecological research. Across short geographical distances, there is substantial variation in the environmental conditions as one moves upslope, driven by the universal temperature lapse rate, viz. a decrease by about 5.6 °C of annual mean temperature per 1000 elevation meters [4]. Because of the small spatial scale of elevational gradients (as compared to latitudinal climate gradients) community assembly is far less constrained by the dispersal limitations of certain species. Rather, organisms are filtered out from largely the same common regional species pool at each elevation according to the matching between their specific habitat requirements and the prevalent environmental conditions [5]. Therefore, the assembly of local communities along elevational gradients is expected to more strongly mirror extant environmental variation rather than biogeographical and historical constraints. This is particularly expected in organisms that have substantial dispersal capacity, but at the same time are not as dispersive that one might encounter them everywhere in a landscape. Many insects capable of flight share exactly these characteristics.

Recent studies have demonstrated predation pressure on insects to decrease consistently along tropical mountains, from a high risk of being consumed at low elevations to a more relaxed predation risk at high elevations [6,7]. Conversely, climatic constraints exert stronger filter functions at high elevations, whereas biotic interactions are generally more important in shaping communities at lower elevations [8], which are usually also far more species-rich. Against this background, one might expect an elevational gradient in the expression of a certain defensive trait at the community level if dealing with a group of focal organisms whose member species show a sufficiently broad diversity in the expression of that particular trait.

**Figure 1:**
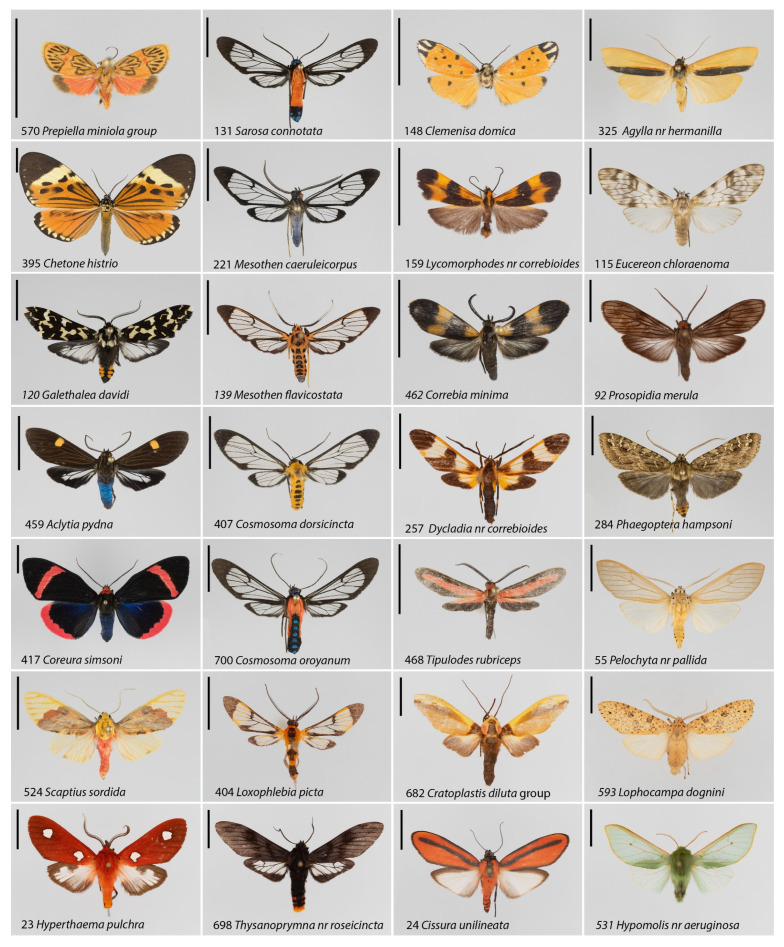
Twenty-eight exemplar species of Arctiinae species (with database numbers, as in Supplementary Table S1) from the Ecuadorian Andes, representing aposematic coloration (first column), hymenopteran mimicry (second column), beetle mimicry (third column) and cryptic appearance (fourth column). Scale bars: 1 cm.

Tiger and lichen moths (Erebidae-Arctiinae) are a species rich clade of moths that lend themselves for this type of study [9]. Especially in the Neotropical realm, Arctiinae moths are highly diverse, also at the local community level [10]. Adult Arctiinae moths are usually nocturnal. Many species show cryptic coloration, usually dull brown, grey or similar. These latter moths employ a strategy of inconspicuousness and background matching with regard to visually hunting predators. In contrast, many other Arctiinae species have wing patterns with colorful bright red, orange, yellow or green-blue spots often contrasting with a dark ground color (Figure 1). Their aposematic appearance is usually coupled with chemical defense, either using secondary plant metabolites sequestered from their larval host plants [11], or collected during the adult stage through pharmacophagy (e.g., [12,13]), or by de novo synthesis of toxic compounds by the insects [3]—yet detailed information is unavailable for most tropical species so far. As a result, colorful tiger and lichen moths are often times unpalatable, or even toxic, to their predators, and they signal their unpalatability through their visual appearance [9], in some cases also supplemented by acoustic signals that address bats as nocturnal predators [2,14]. In many cases, aposematically colored Arctiinae are also part of complex mimicry systems, either Müllerian mimicry rings (where all species are chemically defended and utilize a shared phenotype to signal their unpalatability), or, probably more rarely, in Batesian systems where some colorful species just mimic the warning coloration without being chemically defended [9].

Among Neotropical Arctiinae, three types of visual mimicry systems are common. First, many of these moth species resemble other toxic or unpalatable moths or butterflies from completely unrelated lepidopteran clades, such as Lacturidae, Zygaenidae, Nymphalidae or Notodontidae [9]. Second, many Arctiinae have transparent narrow glassy wings and colorful abdomens, thus resembling hymenopterans such as bees and wasps; hence the vernacular name ‘wasp moths’ for many of these [15]. Bees and wasps are often able to inflict painful stings when attacked. Therefore, hymenopteran mimicry is a potentially powerful strategy for a moth to reduce predation risk. Third, a few Neotropical Arctiinae species are look-alikes of chemically defended beetles in families such as Lycidae, Lampyridae and Chrysomelidae [16,17,18,19]. Additionally, we tested whether body size of tiger moths increases with elevation, as demonstrated along an elevational gradient in Costa Rica by [20].

We here use a sizeable sample of Arctiinae moths collected along a forested elevational gradient in the tropical Andes of southern Ecuador, to assess whether a gradient exists in warning colorations at the community level. Specifically, we test the following hypotheses:

1. In line with an expected decrease in the predation pressure exerted by visually hunting insectivores, the overall prevalence of aposematic coloration continually decreases from low to high elevations.
2. Similarly, the prevalence of hymenopteran and beetle mimicry decreases towards higher elevations.
3. This elevational pattern is largely concordant between all types of warning colorations.
4. Body size of tiger moths increases with elevation.

## 2. Materials and Methods

We sampled moths at 22 sites in southern Ecuador (Province Zamora-Chinchipe), all situated in old-growth forest, at elevations ranging from 1040 to 2677 m a.s.l. These moth samples were originally assembled during an earlier intense biodiversity study along an elevational gradient in South Ecuador [21,22,23] and are here re-analyzed under a new perspective. Our sampling covered 11 elevational bands, each represented by two replicate light-trapping sites. The lowest two sites were situated near the Bombuscaro entrance of Podocarpus National Park at 1040 m. The next two sites were at an elevation of 1380 m in a remnant of disturbed old growth forest near the old road between the provincial capitals of Loja and Zamora. All other sites, at elevations from 1800 to 2677 m with site pairs spaced by steps of approximately 100 elevation meters, were situated in the privately owned Reserva Biológica San Francisco (RBSF), which since 2007 has formed a part of the UNESCO Podocarpus–El Condor Biosphere Reserve. A full list of geographical coordinates and elevations of sampling sites and a map was provided by [24].

According to the classification in [25] the lowest four sites were situated in premontane forest. The next 16 sites at elevations between 1800–2387 m a.s.l. were in evergreen lower montane forest (Types I–III). Finally, four sites at elevations of 2524–2677 m a.s.l. were located in evergreen upper montane forest, which in the study area is represented by a unique formation dominated by one single species of small trees, viz. *Purdiaea nutans* (Clethraceae) [26]. While at the lowest sites the forest has multiple layers and the tallest trees reach 40 m and higher, forest stands become ever lower, with more slender stems, lower basal area, denser herb layer, and increasing canopy openness towards higher elevations [27]. At the highest sites, trees were usually less than 8 m tall and canopy openness was 30% and above. In the study region, the tree line is situated at an unusually low elevation of approximately 3000 m a.s.l. [25,28]. Further information on climate, topography and ecology of the study area can be found in [29].

Moths were attracted to a ‘light tower’ placed at ground level (a gauze cylinder, 1.60 m high, diameter 0.80 m), equipped with two battery-driven 15 W fluorescent tubes (Sylvania blacklight-blue F 15W BLB, and Philips TLD 15 W 05). We sampled moths manually from the illuminated gauze between 6:30 to 9:30 p.m. local time (see [30] for a detailed description of sampling procedures). Moth sampling at each site occurred at least twice during three field campaigns in April and May 1999, between October 1999 and January 2000, and in October and November 2000. If combined samples from two nights per site were still smaller than 80 individuals, additional samples were taken until for each site an aggregated sample of at least 80 Arctiinae moths was available. For some sites at mid-elevations (1800–2400 m a.s.l.) with particularly dense forest vegetation this required up to nine nightly replications [21]. Altogether, 130 light-trapping units were performed to assemble the data presented below.

All Arctiinae specimens arriving at the light tower were collected and subsequently processed in the laboratory to allow their taxonomic identification or at least morpho-species sorting. Identification was primarily achieved by comparison with reference collections held in large natural history museums (see acknowledgements), supplemented by using illustrations of identified moth vouchers on websites. For the purpose of this study, all tentative identifications that formed the basis of earlier analyses of species diversity in the elevational gradient [21,22,23] were revalidated against photographs of type material. An illustrated checklist of all Arctiinae species sampled in southern Ecuador is in preparation and will be published elsewhere. All sampled specimens were permanently deposited in the Staatliches Museum für Naturkunde, Stuttgart, Germany.

Moth species were scored by visual inspection for their type and extent of defensive coloration. A moth species was categorized as aposematic (score = 1) if there are areas on the wings, thorax, and/or abdomens colored in bright yellow, red, orange, or blue. All other species, usually cryptically colored in brown, green, whitish or grey, received a score of 0. Species embedded in mimicry rings involving wasps, bees, or beetles were also scored as aposematic. Warning colors in moths are sometimes hidden when at rest, for example if they occur exclusively on the hind wings. In a supplementary approach we therefore attributed an intermediate graded score of 0.5 to all species where aposematic color pattern elements exist, but are small in extent and are covered by the forewings when the moth is at rest during daytime. However, the community weighted means obtained through these two complementary scoring approaches were highly correlated across the 22 moth community samples (r = 0.991, *p* < 0.0001). We therefore report below only the results from the binary scoring as aposematic versus cryptic.

We attributed a species as showing wasp or bee mimicry if there are substantial transparent areas on the wings, often supplemented by brightly colored stripes on the head, thorax, or abdomen. Some species even possess noticeably wasplike body shapes, and all hymenopteran mimics have narrow elongate wing shapes [31]. Hymenopteran mimicry was scored similarly to aposematism with either 0 (not present) or 1 (present). Wasp or bee mimics occurred almost exclusively in the subtribe Euchromiina as circumscribed by [32]; *Thysanoprymna* nr. *roseocincta* (Phaegopterina) was the only wasp mimic observed in another subtribe (Figure 1).

Thirdly, moths were scored in a category on their own if their external appearance to the human observer suggests a mimicry relationship with beetles in families such as Lycidae, Lampyridae or Chrysomelidae. Cases of putative beetle mimicry were overall rare in our samples, but occurred in unrelated genera such as *Cisthene*, *Clemensia*, *Lycomorphodes*, *Rhabdatomis* (Lithosiinae), *Aemilia*, *Cissura*, *Cratoplastis* (Phaegopterina), *Correbia*, *Correbidia*, *Dycladia*, *Leucotmemis* (subtribe Euchromiina), as well as *Tipulodes* (Ctenuchina). Some exemplar species illustrating various types of cryptic or warning coloration among the Arctiinae sampled in southern Ecuador are shown in Figure 1. We also measured forewing length (from the wing insertion at the mesothorax to the apex) from digital scaled photographs of all species, as a proxy for moth body size. A full list of all species, unique database numbers, individual counts per site, their forewing length and their wing coloration scorings is provided in Supplementary Table S1.

Using the scores of aposematism, hymenopteran mimicry, and beetle mimicry, forewing length and the species × sites abundance matrix, we calculated community weighted means (CWMs hereafter) for these traits of the Arctiinae assemblages at each of the 22 localities. In brief, CWMs denote the average value any trait attains within a community, i.e., the trait value one expects to encounter when drawing a random individual from that community. The analysis of CWMs has proven to be a valuable and powerful tool for analyzing light-trap samples of moths along environmental gradients [33,34]. We also calculated the contribution of the tribe Lithosiini and of five Arctiini subtribes (Euchromiina, Ctenuchina, Phaegopterina, Arctiina, Pericopina) to each local assemblage. Data for Pericopina and Arctiina were not further analyzed statistically due to their low representation (no observations at >50% of the sites, and otherwise accounting for less than 5% of individuals at all other sites).

We then tested for relationships between CWMs of traits and elevation of sampling sites, using standard correlation analysis as implemented in the software package PAST 4.07 [35].

## 3. Results

We sampled 4466 individuals of Arctiinae moths representing 353 species. Sample size varied across light-trapping sites from 85 to 711 individuals (mean: 203.0 per site; SD = 140.4). Altogether, 212 species (representing 1806 individuals) were scored as aposematic, 43 as hymenopteran mimics (609 individuals), and 14 species as beetle mimics (73 individuals) (Table 1). The incidence of aposematic species and mimics strongly varied between subtribes. For example, while all 35 Euchromiina species are hymenopteran mimics, few or none occurred in other subtribes. On the other hand, the highest proportion of cryptic species was found in Lithosiini (83%), followed by Phaegopterina (44%) whereas most species were aposematic in the other groups. Details are provided in Table 1.

**Figure 2:**
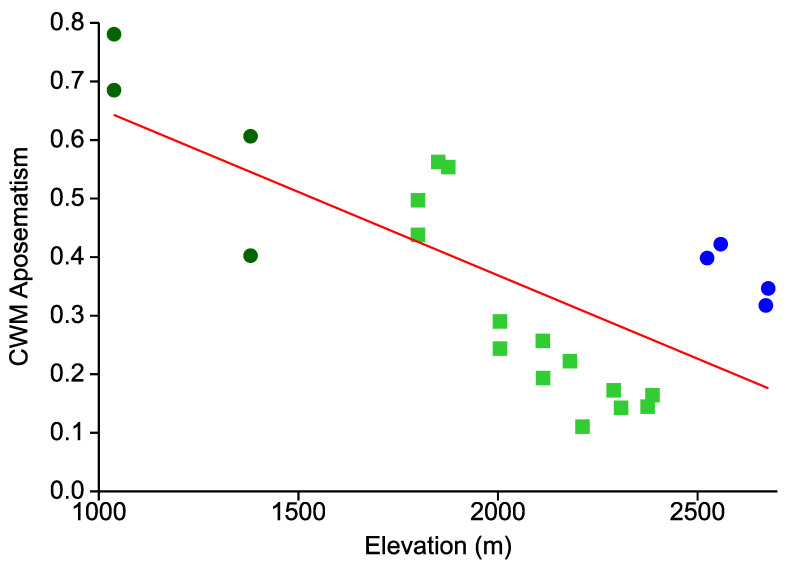
CWMs (Community Weighted Means) of aposematic coloration among 22 local Arctiinae assemblages in an elevational gradient in the Andes of southern Ecuador. Dark green dots: premontane forest; lime green squares: lower montane forest; blue filled circles: upper montane *Purdiaea nutans* forest. Forest types follow [22]. The red line represents an OLS regression.

The communitywide prevalence of aposematism decreased drastically from low towards high elevation sites (r = −0.715, r^2^ = 0.520; *p* = 0.0003; Figure 2). At the two lowest sites, more than two thirds of all Arctiinae individuals were warningly colored. The lowest prevalence (30% and less) of aposematic tiger and lichen moths was observed in the dense lower montane forest stands between 2000–2400 m a.s.l. Only at the four highest sites in the open *Purdiaea nutans* forest did the incidence of aposematic coloration become higher again (around 40–50%).

**Figure 3:**
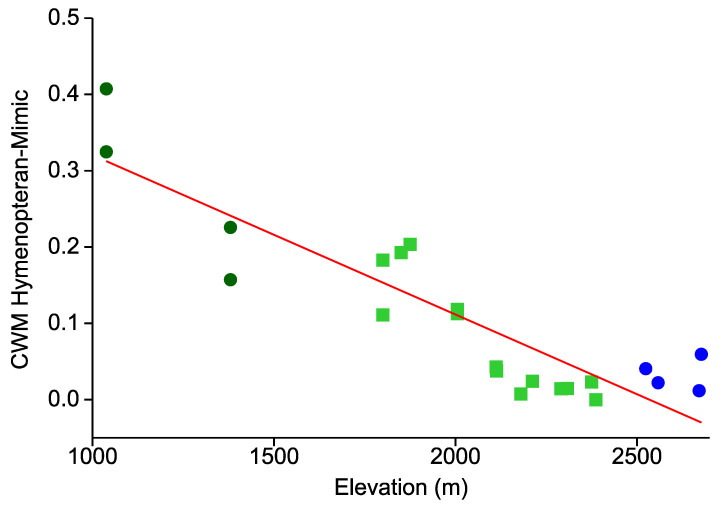
CWMs of hymenopteran mimicry among 22 local Arctiinae assemblages in an elevational gradient in the tropical Andes of southern Ecuador. Dark green dots: premontane forest; lime green squares: lower montane forest; blue filled circles: upper montane *Purdiaea nutans* forest. The red line represents an OLS regression.

Representation of hymenopteran mimics in the local Arctiinae assemblages revealed a similar elevational pattern (Figure 3). Their decline with elevation was even stronger than for all cases of aposematism combined (r = −0.895; r^2^ = 0.801; *p* < 0.0001). At the two Bombuscaro sites the incidence of hymenopteran mimicry was particularly high (30% and above). In contrast, very few wasp or bee mimics (<10% of local assemblages) occurred in dense montane forest at elevations above 2100 m a.s.l., with not even a single case recorded at one site situated at 2387 m amongst a sample of 128 moths from 46 species. The slopes for elevational decline were not significantly different between wasp or bee mimics and warningly colored Arctiinae in general (ANOVA: F = 1.305, *p* = 0.260).

**Figure 4:**
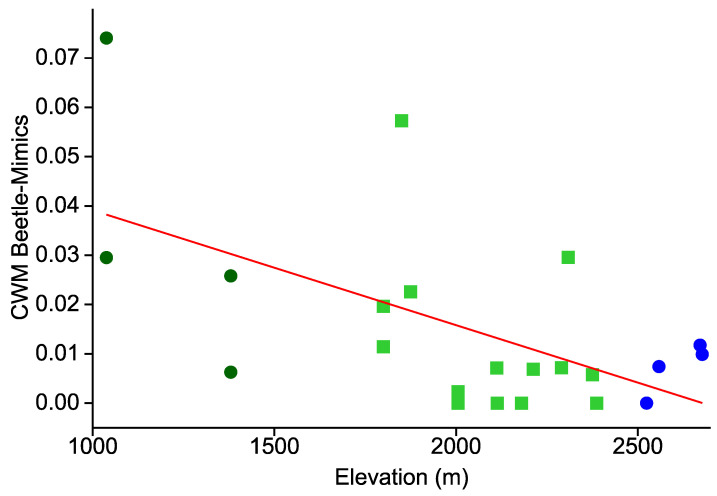
CWMs of beetle mimicry among 22 local Arctiinae assemblages in an elevational gradient in the Andes of southern Ecuador. See Figure 2 for further explanations.

The incidence of beetle mimicry revealed a different spatial pattern (Figure 4). Overall, such phenotypes were rare at our study sites, accounting for but 1.6% of the total Arctiinae moth sample. Still, even this small sample revealed a statistically significant elevational decline (r = −0.584; r^2^ = 0.341; *p* = 0.005). However, this decline was significantly less steep than with aposematic or hymenopteran mimics (F = 57.77, *p* < 0.0001), and there was more scatter in the sparse data. At five of 22 sites we did not observe any beetle mimics.

**Figure 5:**
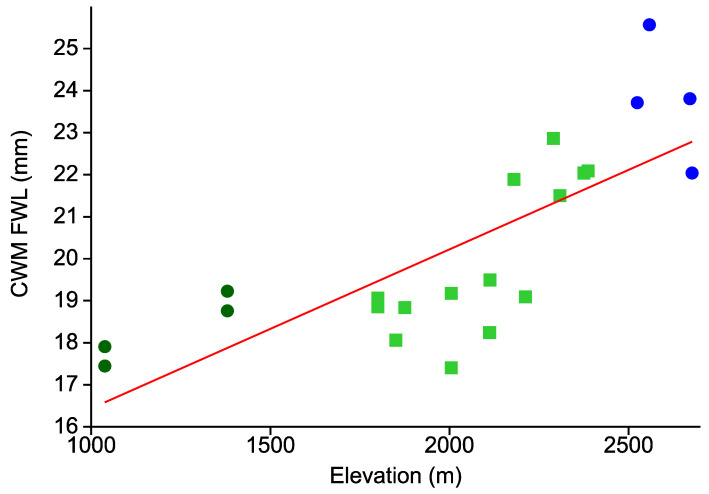
CWMs of forewing length (as a proxy of body size) among 22 local Arctiinae see Figure 2 for further explanations.

Overall, the average body size of the Arctiinae species represented in the local assemblages substantially increased along the elevational gradient (Figure 5). While at the two Bombuscaro sites mean forewing length was below 18 mm, CWMs per site ranged from 22–25.5 mm in upper montane *Purdiaea nutans* forest. This pattern was again highly significant (r = 0.770, r^2^ = 0.593, *p* < 0.0001).

The observed elevational changes in eco-morphological traits such as warning coloration, mimicry types and forewing length coincided with intense turnover in the gross taxonomic composition of local Arctiinae assemblages. In particular, the contribution of Euchromiina (r= −0.868; r^2^ = 0.753; *p* < 0.0001) and Ctenuchina (r = 0.638, r^2^ = 0.407, *p* < 0.002) decreased substantially towards higher elevations, while the prevalence of Lithosiinae increased (r = 0.658; r^2^ = 0.433, *p* < 0.002). Phaegopterina, in contrast, revealed no significant elevational pattern (r = 0.340, r^2^ = 0.116, *p* = 0.121).

## 4. Discussion

According to our surveys, the incidence of warning coloration among megadiverse tropical Andean Arctiinae moths continually and strongly decreased towards higher elevations. This pattern is in line with expectations based on studies in other tropical realms which indicated that predation pressure, especially by insectivorous birds, declines with elevation [36,37]. Warning colors always come with a cost. This not only includes the production of the relevant pigments, but also mortality risks due to attacks, for example by inexperienced predators. Therefore, aposematism only pays off if the insects, which are conspicuous to their enemies, are chemically defended and thus unpalatable. Alternatively, their toxic models must be sufficiently common in case of Batesian mimicry rings. To achieve unpalatability status, many Arctiinae moths require access to toxic plant species either during their larval or adult stages. Since the richness of vascular plants decreases distinctly with elevation also in the study region in the Andes [38], accompanied by changes in many functional plant attributes [39], chances to sequester such defensive plant metabolites become ever smaller upslope (see [40,41] for case studies on elevational gradients in phytochemistry in the European Alps). Hence, it is likely that the gradient in warning coloration found in the Andes also reflects a similar elevational gradient in the sequestration of plant-derived defenses. This was exemplified for pyrrolizidine alkaloid containing plants such as *Prestonia* (Apocynaceae) [12] which only occurred at the lowermost sites of the elevational gradient.

Our studied elevational gradient, though incomplete, spanned a substantial range of vegetation types and climatic conditions. Therefore, we expected to observe gradients in species attributes along these stark ecological contrasts. However, even along much less extensive topographic gradients in the lowland tropical forest, distinct variation in the extent of aposematism and mimicry has recently been reported from Costa Rica [33]. Hence, the perspective on communitywide patterns in traits such as defense against visually hunting predators could become a rewarding paradigm that is accessible from the morphological study of samples alone, without the need for experimentation, which may often not be feasible in species-rich tropical conservation areas, due to the rarity and unknown life histories of most component species.

It should be noted here that some Arctiinae moths not only use visual signals that are predominantly effective against diurnal predators, such as most birds, lizards and alike, but also use acoustic signals or sonar jamming to advertise their unpalatability towards nocturnally hunting bats. Obviously, we could not score the capacity of the moth species at our study sites in that regard based on collection specimens. A recent report suggests that to some extent acoustic defenses of tiger moths can be predicted from the morphology of the moth’s tymbal organs [42]. Since all material is preserved in museum collections, a thorough anatomical re-examination of all species would be possible in future studies. In the present analysis, however, we focused on the visual appearance of the moths.

The incidence of aposematism and hymenopteran mimicry was particularly high at the lowest sites, situated at the foothills of the Cordillera Real. We infer from this pattern that in premontane forest predation pressure on moths is likely most intense. This would be expected, given the immense species diversity of tropical rainforests across all trophic levels and the complexity of their food webs.

Unfortunately, neither for insectivorous birds nor for bees and wasps that might serve as models for hymenopteran mimics do we have precise data on their elevational distribution or abundance patterns in the study region. However, insectivorous birds generally decline in abundance and richness along tropical Andean elevational gradients [43,44]. Inside our core research area, the RBSF reserve, richness of insectivore birds peaked around 2000 m asl and distinctly declined towards 2600 m [37]. Hence, the parallel decline of aposematism and hymenopteran mimicry does not come as a surprise. Beetle mimics were overall much rarer amongst moths, but also declined in abundance towards higher elevations. For most beetle families that may function as models no abundance or diversity data are available from the region. However, for leaf beetles a hump-shaped peak in species richness around 2000 m elevation was observed, followed by a steep decline in upper montane forest [45]. Our own (unpublished) qualitative observations of beetles (e.g., longhorn and scarabeid beetles) attracted to UV lamps along Andean elevational gradients in Ecuador and Peru likewise suggest that overall beetle diversity indeed generally strongly declines with elevation.

For all three types of warning coloration we observed lowest incidences in the very dense lower montane forest inside the RBSF reserve, at elevations between 2000–2400 m. We interpret this pattern to reflect the low visibility of warning signals to potential predators, whereas in the more open upper montane *Purdiaea nutans* forest the incidence of warning colors and hymenopteran mimicry again increased slightly. This might indicate that predation pressure by visually oriented insectivores such as birds is also higher here.

Body size showed the opposite trend than aposematism: While the latter significantly decreased, body size significantly increased towards upper montane forest. Brehm et al. [20] discussed potential mechanisms favoring larger size at higher elevations, such as different physical requirements to wings due to a thinner atmosphere, but also a higher predation risk at low elevations that selects for smaller species. It is particularly remarkable that small and aposematic Lithosiini moths exclusively occurred at the lowermost sites of the study area, being replaced by mostly whitish and larger *Agylla* species at higher elevations. A very similar pattern was observed in Costa Rica [20].

Our study region represents a global biodiversity hotspot for many plant as well as animal groups [46]. Even though our study sites are situated inside conservation areas, pressures continue on Andean forests in southern Ecuador, especially through illegal logging or burning to establish new pasture sites [47]. These pressures are highest at the low elevations where human settlements and economic activities are also concentrated. Hence, especially those forest types which were shown to harbor the highest incidence of warning colorations experience the highest risk of being further reduced in area or even lost.

Another current threat to tropical mountain biodiversity emerges from global climate change. Studies on Mount Kinabalu in SE Asia revealed that moth faunas have shifted upslope significantly within about 50 years [48]. Even though some of these observed shifts might also be due to seasonal fluctuations [49], tropical mountain biota are certainly under pressure through climate change [4,50]. In the light of these threats, especially again the moth communities of low elevations, characterized by the highest levels of biotic defense, will need to move upslope, but it remains to be tested whether the plants from which they sequester their defenses will be able to migrate at the same pace.

We collected the moths from which we obtained our data over 20 years ago. In the meantime, climate change as well as land-use pressures have continued in the region [47]. It would be therefore rewarding to resurvey the sites again to check how far moth species have indeed started to move upslope and whether this might also have changed the ways in which these organisms are embedded in the food webs of their ecosystems. Analyzing spatial patterns of defensive coloration could be an interesting and cost-effective tool in that regard.

## Figures and Tables

**Table 1:** Number of species scored as aposematic, as hymenopteran mimic, as beetle mimic and as cryptic. Percentages represent the incidence of aposematism and crypsis within each taxon.

Taxon	Species	Cryptic	Aposematic		
				Wasp Mimics	Beetle Mimics
Lithosiini	66	55 (83%)	11 (17%)	0	4
Arctiini					
Arctiina	7	1 (14%)	6 (86%)	0	0
Ctenuchina	71	13 (18%)	58 (82%)	7	6
Euchromiina	35	0	35 (100%)	35	0
Pericopina	13	0	13 (100%)	0	0
Phaegopterina	161	72 (45%)	89 (55%)	1	4
Sum Arctiinae	353	141 (40%)	212 (60%)	43	14

## Data Availability

All data used for the analyses presented here are available in the Supplementary Table S1.

## Supplementary Materials

The following are available online at https://www.mdpi.com/article/10.3390/insects12100903/s1, Table S1: Tabular compilation of all 353 Arctiinae species represented in samples from 22 light trap sites in South Ecuador, with measurements of forewing length, status of their aposematic coloration, inclusion in mimicry rings with hymenopterans or beetles, and the observed numbers of specimens per site. Likewise included is the elevation of each light trap site and a unique database ID number for each moth species. The old ID refers to the morphospecies sorting used in earlier publications derived from these moth samples.
